# Novel circulating lipid measurements for current dyslipidemias in non-treated patients undergoing coronary angiography: PCSK9, apoC3 and sdLDL-C

**DOI:** 10.18632/oncotarget.12471

**Published:** 2016-10-04

**Authors:** Sha Li, Xi Zhao, Yan Zhang, Cheng-Gang Zhu, Yuan-Lin Guo, Na-Qiong Wu, Rui-Xia Xu, Ping Qing, Ying Gao, Jing Sun, Geng Liu, Qian Dong, Jian-Jun Li

**Affiliations:** ^1^ Division of Dyslipidemia, State Key Laboratory of Cardiovascular Disease, Fu Wai Hospital, National Center for Cardiovascular Diseases, Chinese Academy of Medical Sciences, Peking Union Medical College, Beijing, China

**Keywords:** proprotein convertase subtilisin/kexin type 9, apolipoprotein CIII, small dense low density lipoprotein cholesterol, dyslipidemia, discordance

## Abstract

Plasma levels of proprotein convertase subtilisin/kexin type 9 (PCSK9), apolipoprotein C-III (apoC3) and small dense low density lipoprotein cholesterol (sdLDL-C), have been recently recognized as circulating atherosclerosis-related lipid measurements. We aimed to elucidate their associations with current dyslipidemias, and identify their levels at increased risk to dyslipidemia. A total of 1,605 consecutive, non-treated patients undergoing diagnostic/interventional coronary angiography were examined. Plasma PCSK9 and apoC3 levels were determined using a validated ELISA assay, and sdLDL-C was measured by the Lipoprint LDL System. Plasma levels of PCSK9, apoC3, and sdLDL-C were associated with the current dyslipidemias classification (all *p*<0.001). PCSK9 significantly conferred prediction of both hypercholesterolemia and combined hyperlipidemia at a level of 235 ng/ml; apoC3 levels for hypertriglyceridemia, hypercholesterolemia and combined hyperlipidemia were 80.0, 71.5, and 86.4 g/ml, respectively; and sdLDL-C for hypertriglyceridemia, hypercholesterolemia, combined hyperlipidemia and hypo high density lipoprotein (HDL) cholesterolemia 3.5, 2.5, 4.5, and 2.5 mg/dl, respectively (all *p*<0.001 for area under the receiver-operating characteristic curve). In a polytomous logistic model comparing increasing LDL-C categories, the interactions with high PCSK9, apoC3, and sdLDL-C elevated gradually. Similarly, apoC3 and sdLDL-C showed elevated interaction with increased triglyceride categories, and only sdLDL-C showed interaction with decreased HDL cholesterol (HDL-C) categories. Furthermore, discordances of PCSK9, apoC3, and sdLDL-C with current dyslipidemias were observed. PCSK9, apoC3, and sdLDL-C showed significant interactions with current dyslipidemias, and were predictive in the screening. The substantial discordances with current dyslipidemias might provide novel view in lipid management and further cardiovascular benefit.

## INTRODUCTION

Dyslipidemias, the recognized risk factors for atherosclerotic cardiovascular disease (ASCVD) [1–3], are defined as an abnormal lipid profile including elevated triglycerides (TG), low-density lipoprotein (LDL) cholesterol (LDL-C), and low high-density lipoprotein (HDL) cholesterol (HDL-C). In addition, other lipoprotein classes or lipid regulators, whose plasma levels can not be improved significantly or even be increased by statin treatment, play important roles in determining cardiovascular risk [4–6].

In recent years, a wealth of information from biological or genetic researches on lipid metabolism has led to the identification of several markers, that may be targeted to improve lipid profiles and possible cardiovascular risk in patients with dyslipidemias. However, the measurement of these lipids has not been fully adopted in medical and complete medical checkups. Notably, novel circulating atherosclerosis-related lipid measurements including proprotein convertase subtilisin kexin type 9 (PCSK9) as a key regulator of circulating LDL-C [7]; apolipoprotein C-III (apoC3) as a critical modulator of TG metabolism [8]; and small dense LDL-C as the very atherogenic subspecies of LDL [9]; have been developed with a growing number of evidence, respectively. These measurements could add significant information about lipid condition and contribute to the assessment of cardiovascular risk to the standard lipid profile in clinical practice.

Despite this knowledge on the associations of PCSK9, apoC3, and sdLDL-C with many of current lipid parameters and on their potential roles in cardiovascular health, the associations of these novel lipid measurements with current dyslipidemias classification and their combined effects have not been explored and identified so far. It was, therefore, the purpose of the present study to investigate the interactions of PCSK9, apoC3, and sdLDL-C with current dyslipidemias, and identify their levels in predicting dyslipidemias in a large cohort of Chinese non-treated patients undergoing coronary angiography (CAG).

## RESULTS

### Baseline characteristics

Basic characteristics of the study population were summarized in Table 1. A total of 1605 patients enrolled in the analysis, of which, 359 (22.4%) patients were hypertriglyceridemia, 250 (15.6%) were hypercholesterolemia, 312 (19.4%) were combined hyperlipidemia, 323 (20.1%) hypo HDL cholesterolemia, and 361 (22.5%) were normalipidemia. Median age of study participants was 55.5±11.2 years and 995 (62.0%) were men. Patients with combined hyperlipidemia presented the youngest age (54.1±11.0 years), and hypo HDL cholesterolemia showed the highest prevalence of men (77.7%) compared to other dyslipidemia groups (*p* < 0.001).

**Table 1:** Baseline characteristics of the study patients according to the current dyslipidemias

Variables	Hyper triglyceridemia	Hyper cholesterolemia	Combined hyperlipidemia	Hypo HDL cholesterolemia	Normalipidemia	*P*-value
Total						
Number	359	250	312	323	361	-
Prevalence,%	22.4	15.6	19.4	20.1	22.5	-
Men,%(n)	66.9(240)	48.8(122)	60.9(190)	77.7(251)	53.2(192)	**<0.001**
Age (year)	54.3±10.0	56.3±12.1	54.1±11.0	55.8±11.8	57.2±10.9	**<0.001**
BMI (kg/m2)	26.63±3.42	24.51±3.57	26.43±3.34	25.53±3.62	25.34±13.34	**<0.001**
Hypertension,%(n)	57.9(208)	51.2(128)	58.7(183)	53.9(174)	54.8(198)	**0.354**
Diabetes mellitus,%(n)	19.5(70)	17.2(43)	26.3(82)	19.5(63)	15.0(54)	**0.005**
Current smoking	37.6(135)	20.8(52)	38.8(121)	35.3(114)	26.3(95)	**<0.001**
						
Laboratory analysis						
TG(mmol/L)	2.62±1.11	1.24±0.30	3.50±2.60	1.26±0.28	1.07±0.31	**<0.001**
TC(mmol/L)	4.40±0.52	5.99±0.88	6.10±0.87	4.10±0.64	4.38±0.52	**<0.001**
HDL-C(mmol/L)	0.91±0.26	1.36±0.40	1.08±0.25	0.87±0.12	1.33±0.30	**<0.001**
LDL-C(mmol/L)	2.70±0.64	4.21±0.96	3.91±1.04	2.78±0.66	2.73±0.57	**<0.001**
ApoAI(g/L)	1.23±0.24	1.53±0.38	1.40±0.29	1.13±0.18	1.43±0.24	**<0.001**
ApoB(g/L)	0.98±0.16	1.24±0.27	1.32±0.28	0.91±0.19	0.87±0.18	**<0.001**
Lp(a)(mg/L)	177.59±206.64	300.14±268.95	214.76±250.56	215.89±231.52	218.60±240.20	**<0.001**
PCSK9 (ng/ml)	231.10±68.58	255.30±70.82	251.99±65.63	221.30±67.03	228.87±67.24	**<0.001**
ApoC3 (μg/ml)	107.24±44.49	85.11±39.53	138.62±72.94	64.17±33.04	67.09±36.07	**<0.001**
sdLDL-C (mg/dl)	10.15±7.30	7.86±9.39	18.37±12.88	4.59±5.30	2.77±4.01	**<0.001**
Glucose(mmol/L)	5.61±1.63	5.57±1.72	6.04±2.36	5.32±1.20	5.48±1.19	**<0.001**
HbA1C(%)	6.06±1.02	6.04±1.07	6.21±1.06	6.01±1.01	5.97±0.92	**0.012**
Hs-CRP(mg/L)	2.53±2.70	2.44±2.88	2.64±3.06	2.47±2.16	2.02±2.63	**0.018**

Data shown are mean ± SD, or %(n). The bold values indicate statistical significance and are bolded to improve the readability of the table. BMI, body mass index; TG, triglyceride; TC, total cholesterol; HDL-C, high density lipoprotein cholesterol; LDL-C, low density lipoprotein-cholesterol; Apo, apolipoprotein; Lp(a), lipoprotein (a); PCSK9, proprotein convertase subtilisin/kexin type 9; sd, small dense; HbA1C, hemoglobin A1C; Hs-CRP, high sensitively C reactive protein.

The mean values of routine laboratory lipid parameters including TG, total cholesterol (TC), HDL-C, LDL-C, apoAI, apoB, lipoprotein (a), and novel lipid measurements including PCSK9, apoC3, and sdLDL-C were presented in Table 1 separately. For comparison, baseline values for glucose, hemoglobin Alc (HbA1C), and high-sensitivity C-reactive protein (hs-CRP) were provided as well.

### Associations of PCSK9, apoC3 and sdLDL-C with current dyslipidemias

Table 2 showed adjusted values of the routine laboratory lipid parameters and novel lipid measurements according to current dyslipidemias in general linear models. Compared with normalipidemia, PCSK9 levels were significantly and independently higher in only hypercholesterolemia and combined hyperlipidemia (228.19±3.60 *vs*. 251.35±4.30 *vs*. 250.05±3.80 ng/ml); apoC3 levels were higher specially in combined hyperlipidemia (137.63±3.18 *vs*. 67.30±3.03 μg/ml), followed by hypertriglyceridemia (107.09±2.86 μg/ml), and hypercholesterolemia (85.95±3.71 μg/ml); sdLDL-C levels were higher in all dyslipidemia groups, the values in hypertriglyceridemia, hypercholesterolemia, combined hyperlipidemia, and hypo HDL cholesterolemia were 10.12±0.54, 8.27±0.71, 18.39±0.60, and 4.62±0.59 mg/dl, respectively (*vs*. 3.05±0.57 mg/dl in normalipidemia).

**Table 2:** Adjusted values of lipid parameters in the current dyslipidemia groups

Lipid parameters	Hyper triglyceridemia	Hyper cholesterolemia	Combined hyperlipidemia	Hypo HDL cholesterolemia	Normalipidemia	*P*-value
TG(mmol/L)	2.60±0.07	1.29±0.08	3.44±0.07	1.26±0.07	1.12±0.07	**<0.001**
TC(mmol/L)	4.41±0.04	5.98±0.04	6.09±0.04	4.11±0.04	4.37±0.04	**<0.001**
HDL-C(mmol/L)	0.92±0.01	1.34±0.02	1.09±0.02	0.88±0.02	1.30±0.01	**<0.001**
LDL-C(mmol/L)	2.71±0.04	4.24±0.05	3.92±0.04	2.79±0.04	2.75±0.04	**<0.001**
ApoAI(g/L)	1.24±0.01	1.51±0.02	1.40±0.02	1.14±0.02	1.40±0.01	**<0.001**
ApoB(g/L)	0.98±0.01	1.25±0.01	1.32±0.01	0.91±0.01	0.88±0.01	**<0.001**
Lp(a)(mg/L)	180.06±12.74	298.75±15.52	216.18±13.3	220.06±13.47	215.23±13.01	**<0.001**
PCSK9 (ng/ml)	231.78±3.53	251.35±4.30	250.05±3.80	225.98±3.73	228.19±3.60	**<0.001**
ApoC3 (μg/ml)	107.09±2.86	85.95±3.71	137.63±3.18	64.54±3.16	67.30±3.03	**<0.001**
sdLDL-C (mg/dl)	10.12±0.54	8.27±0.71	18.39±0.60	4.62±0.59	3.05±0.57	**<0.001**

A general linear model was used to study the lipid measurements over the groups of dyslipidemias, with adjustments for confounding factors including age, gender, BMI, hypertension, HbA1C, smoking, hs-CRP, and coronary statues, and to calculate the adjusted mean values. The bold values indicate statistical significance and are bolded to improve the readability of the table. TG, triglyceride; TC, total cholesterol; HDL-C, high density lipoprotein cholesterol; LDL-C, low density lipoprotein-cholesterol; Apo, apolipoprotein; Lp(a), lipoprotein (a); PCSK9, proprotein convertase subtilisin/kexin type 9; sd, small dense.

**Figure 1:**
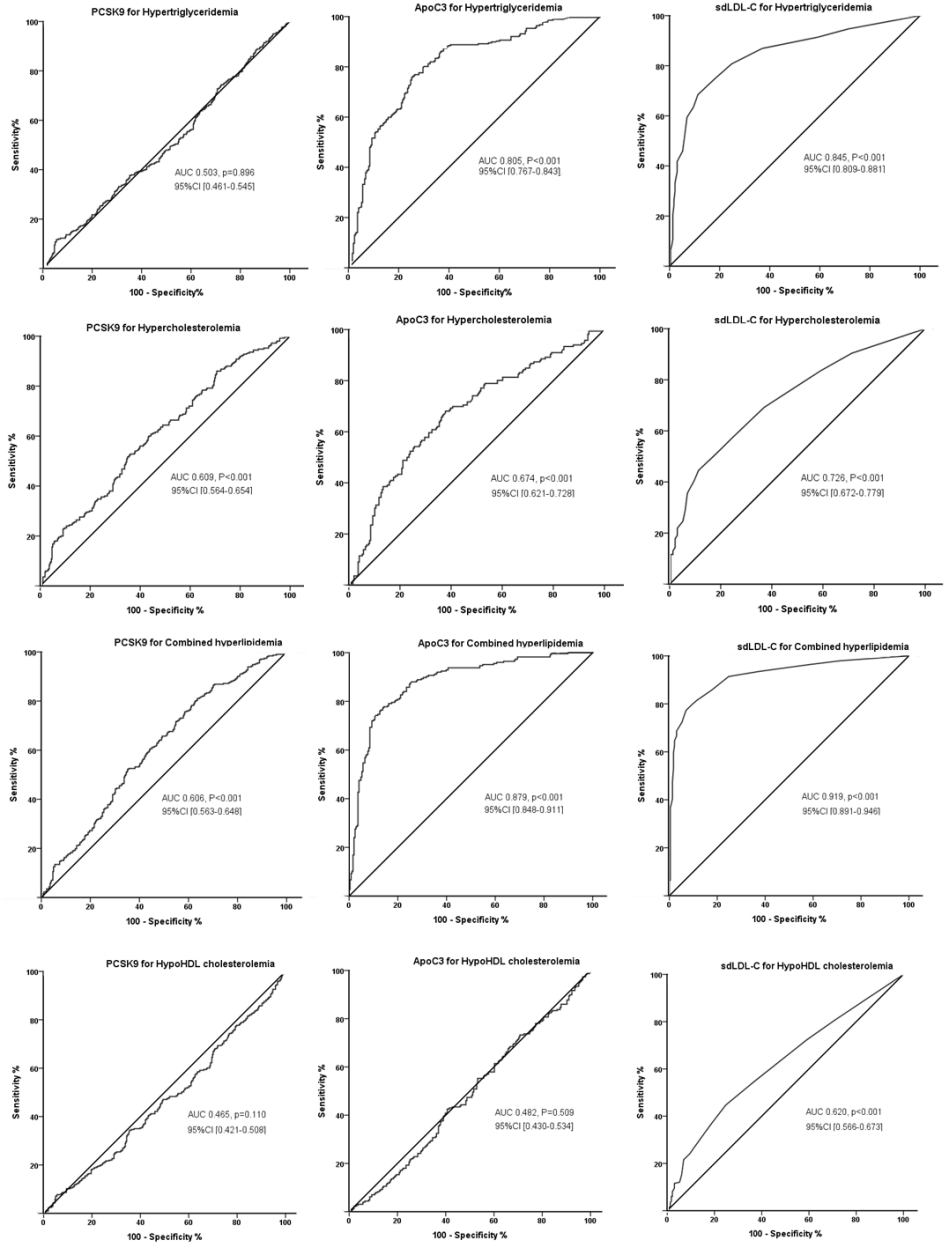
ROC curves of PCSK9, apoC3, and sdLDL-C for predicting the presence of current dyslipidemias Each of PCSK9, apoC3, and sdLDL-C level for predicting hypertriglyceridemia, hypercholesterolemia, combined hyperlipidemia and hypo HDL cholesterolemia were evaluated, and the area under curve (AUC) values and 95%CI were shown.

Furthermore, receiver operating characteristic (ROC) analysis was performed to test the accuracy of each of the 3 lipid parameters for predicting the presence of the current lipid disorder (Figure 1). As shown in Figure 1, the values of the area under the ROC curve (AUC) for predicting hypertriglyceridemia were most significant by sdLDL-C (0.845, 95% CI 0.809-0.881, *p* < 0.001), followed by apoC3 (0.805, 95% CI 0.767-0.843, *p* < 0.001). For predicting hypercholesterolemia, the values of AUC remained the highest by sdLDL-C (0.726, 95% CI 0.672-0.779, *p* < 0.001), followed by apoC3 0.674, 95% CI 0.621-0.728, *p* < 0.001) and PCSK9 (0.609, 95% CI 0.564-0.654, *p* < 0.001). For predicting combined hypercholesterolemia, the values of AUC by sdLDL-C, apoC3, and PCSK9 were 0.919, 95% CI 0.891-0.946; 0.879, 95% CI 0.848-0.911; and 0.606, 95% CI 0.563-0.648; respectively (all *p* < 0.001). For predicting hypo HDL cholesterolemia, however, only sdLDL-C showed the significant value (0.620, 95% CI 0.566-0.673, *p* < 0.001). PCSK9 significantly conferred prediction of both hypercholesterolemia and combined hyperlipidemia at levels of 235 ng/ml; apoC3 levels for hypertriglyceridemia, hypercholesterolemia and combined hyperlipidemia were 80.0, 71.5, and 86.4 μg/ml, respectively; and sdLDL-C for hypertriglyceridemia, hypercholesterolemia, combined hyperlipidemia and hypo HDL cholesterolemia were 3.5, 2.5, 4.5, and 2.5 mg/dl, respectively (all *p* < 0.001).

According to the results from ROC analysis, we chosen the cut-points of PCSK9 with 235 ng/ml, apoC3 with 71.5 μg/ml, and sdLDL-C 2.5 mg/dl as the high values of the measurements in the present analysis. In polytomous logistic regression models, we analyzed the interactions of the elevated PCSK9, apoC3, and sdLDL-C levels with increasing dyslipidemias (Table 3). We demonstrate that the magnitude of the interactions increases with increasing LDL-C categories. The adjusted ORs for high PCSK9 increased from 1.22, 95% CI 0.94-1.59 at an 2.59 < LDL < 3.37 mmol/L, to 3.06, 95%CI 2.13-4.39 at an LDL-C > 4.14 mmol/L; for high apoC3 were from 1.08, 95% CI 0.79-1.49, to 2.73 95% CI 1.71-4.34; and for high sdLDL-C were from 1.97, 95% CI 1.38-2.79, to 4.50, 95% CI 2.59-7.82. Increasing interactions of high apoC3 and sdLDL-C with increasing TG categories, and high sdLDL-C with decreasing HDL-C categories were also observed (Table 3).

**Table 3:** Logistic regression analysis for high PCSK9, ApoC3, sdLDL-C with current increasing dyslipidemias

	High PCSK9	*P*-value	HighApoC3	*P*-value	High sdLDL-C	*P*-value
TG≤1.70 mmol/L	Reference		Reference		Reference	
1.7<TG≤2.25 mmol/L	1.11[0.84-1.47]	0.475	6.21[4.18-9.25]	**<0.001**	4.14[2.70-6.34]	**<0.001**
2.25<TG≤5.65 mmol/L	1.03[0.79-1.34]	0.837	21.4[.0–35.3]	**<0.001**	12.2[6.81-21.8]	**<0.001**
TG>5.65 mmol/L	0.98[0.51-1.90]	0.963	Very High	**<0.001**	11.8[1.45-95.9]	**0.021**
						
LDL-C≤2.59 mmol/L	Reference		Reference		Reference	
2.59<LDL-C≤3.37 mmol/L	1.22[0.94-1.59]	0.142	1.08[0.79-1.49]	0.616	1.97[1.38-2.79]	**<0.001**
3.37<LDL-C≤4.14 mmol/L	1.49[1.10-2.02]	**0.009**	1.71[1.18-2.50]	**0.005**	2.68[1.75-4.11]	**<0.001**
LDL-C>4.14 mmol/L	3.06[2.13-4.39]	**<0.001**	2.73[1.71-4.34]	**<0.001**	4.50[2.59-7.82]	**<0.001**
						
HDL-C>1.55 mmol/L	Reference		Reference		Reference	
1.04<HDL-C≤1.55 mmol/L	1.12[0.76-1.67]	0.568	1.18[0.72-1.95]	0.506	1.99[1.14-3.47]	0.016
HDL-C≤1.04 mmol/L	0.82[0.55-1.22]	0.321	1.20[0.72-2.00]	0.491	1.54[1.25-1.89]	**<0.001**

Polytomous logistic regression analysis to evaluate the association between the high values according to the cut-points from the analysis of receiver-operating characteristic curves to explore the strength of the interactions with current dyslipidemias. The cut-points of PCSK9, apoC3, sdLDL-C were 235 ng/ml, 71.5 μg/ml, 2.5 mg/dl, respectively in the present analysis. Odds ratio (OR) and 95% confident interval (95% CI) was calculated with the logistic models adjusted by potential confounding factors such as age, gender, BMI, hypertension, HbA1C, smoking, hs-CRP, dyslipidemias classification, and coronary status. TG, triglyceride; LDL-C, low density lipoprotein-cholesterol; HDL-C, high density lipoprotein cholesterol.

Additionally, we found that either non-significant higher or significant higher levels of PCSK9, apoC3, and sdLDL-C in patients with positive angiographic results than those with negative ones (supplemental Table). When patients were divided according to diseased coronary branches or number of diseased vessels, the levels of PCSK9, apoC3, and sdLDL-C (*vs*. negative angiographic results) also showed the similar associations (supplemental Table).

### Discordances of PCSK9, apoC3 and sdLDL-C with current dyslipidemias

**Figure 2:**
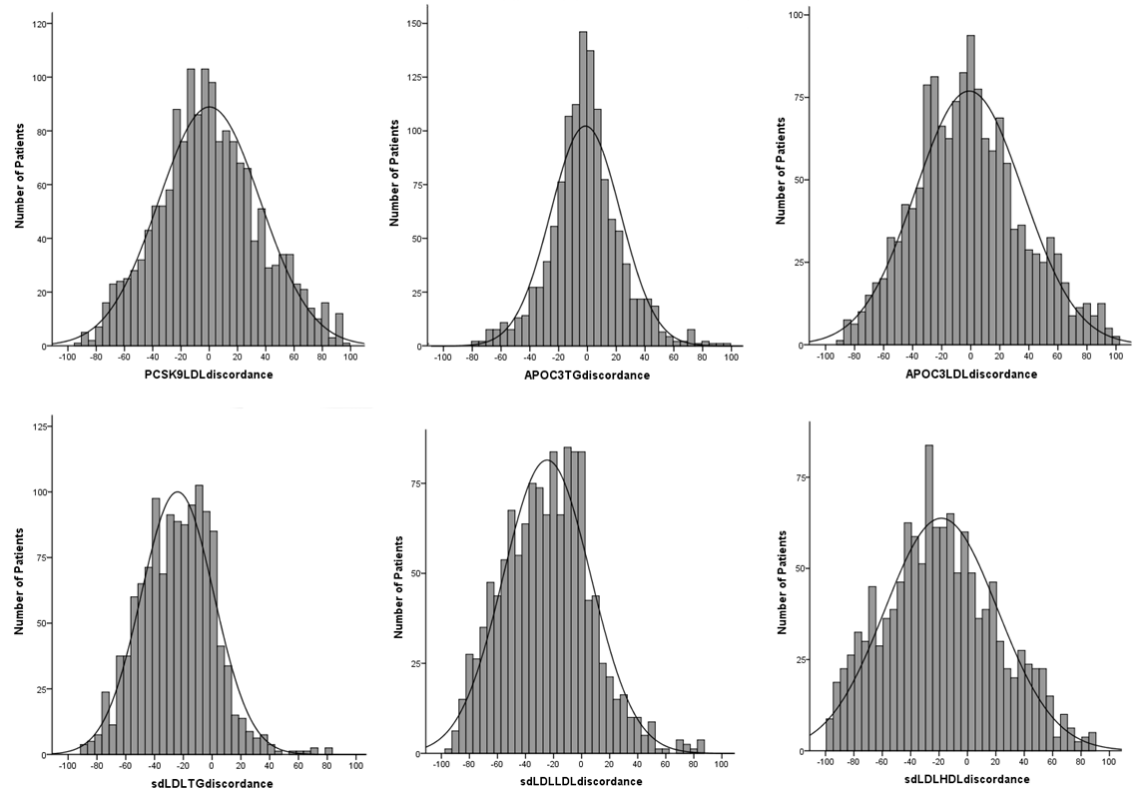
Distributions of discordance scores for PCSK9, apoC3, and sdLD-C to current lipid The discordance scores were the remainders of the precentiles, for example, apoC3/TG discordance score was equal to apoC3 percentile - TG percentile).

Despite the interactions (data shown above), a high degree of discordances of PCSK9, apoC3, and sdLDL-C with current dyslipidemias were detected (Figure 2). As shown in Figure 2, the discordance scores of PCSK9 with LDL-C (PCSK9 percentile - LDL-C percentile), apoC3 with TG or LDL-C (apoC3 percentile - TG or LDL-C percentile) were normally distributed. The discordance scores of sdLDL-C with TG, LDL-C, and HDL-C (sdLDL-C percentile - TG or LDL-C or HDL-C percentile) appeared to be skewed distributions.

According to the kurtosis and skewness of the shading in Figure 2, we considered uniformly the concordance were patients who with discordance scores from -15 to 15, and the either side more than 15 scores were considered to be discordant. As shown in Table 4, substantial discordances with PCSK9, apoC3, and sdLDL-C in each dyslipidemia group were observed. Even in patients with current normalipidemia, the discordances with PCSK9, apoC3, and sdLDL-C could be noted, for example, discordant PCSK9 > LDL-C presented 44.3% in patients with current normalipidemia.

**Table 4:** Prevalence of lipid discordance in the current dyslipidemias

Lipid discordance	Hyper triglyceridemia	Hyper cholesterolemia	Combined hyperlipidemia	Hypo HDL cholesterolemia	Normalipidemia	*P*-value
Discordant ApoC3<TG	41.9%	12.7%	23.1%	22.8%	10.6%	
Concordant ApoC3=TG	53.4%	39.8%	69.3%	61.8%	57.3%	
Discordant ApoC3>TG	4.7%	47.6%	7.6%	15.4%	32.1%	
						**<0.001**
Discordant sdLDL<TG	89.8%	43.2%	66.2%	62.8%	39.1%	
Concordant sdLDL=TG	9.4%	42.5%	29.8%	33.2%	59.6%	
Discordant sdLDL>TG	0.8%	14.4%	4.0%	4.0%	1.3%	
						**<0.001**
Discordant PCSK9<LDL-C	22.0%	55.6%	47.4%	26.6%	22.2%	
Concordant PCSK9=LDL-C	35.7%	36.0%	38.5%	36.5%	33.5%	
Discordant PCSK9>LDL-C	42.3%	8.4%	14.1%	36.8%	44.3%	
						**<0.001**
Discordant ApoC3<LDL-C	15.9%	71.1%	34.2%	40.8%	35.4%	
Concordant ApoC3=LDL-C	25.6%	25.9%	40.0%	34.6%	39.8%	
Discordant ApoC3>LDL-C	58.5%	3.0%	25.8%	24.6%	24.8%	
						**<0.001**
Discordant sdLDL<LDL-C	39.3%	91.1%	57.1%	60.3%	65.2%	
Concordant sdLDL=LDL-C	39.3%	6.8%	32.3%	36.7%	33.9%	
Discordant sdLDL>LDL-C	21.3%	2.1%	10.6%	3.0%	0.9%	
						**<0.001**
Discordant sdLDL<HDL-C	29.5%	74.0%	30.3%	47.7%	97.4%	
Concordant sdLDL=HDL-C	38.9%	17.8%	24.2%	42.7%	2.2%	
Discordant sdLDL>HDL-C	31.6%	8.2%	45.5%	9.5%	0.4%	
						**<0.001**

Data shown are %. The discordance scores were the remainders of the precentiles, for example, apoC3/TG discordance score was equal to apoC3 percentile - TG percentile).The concordance were patients who with discordance scores from -15 to 15, and the either side more than 15 scores were considered to be discordant. The bold values indicate statistical significance and are bolded to improve the readability of the table. TG, triglyceride; LDL-C, low density lipoprotein-cholesterol; HDL-C, high density lipoprotein cholesterol; Apo, apolipoprotein; PCSK9, proprotein convertase subtilisin/kexin type 9; sd, small dense.

## DISCUSSION

In the present study, we, for the first time, addressed the association of 3 novel circulating atherosclerosis-related lipid measurements including PCSK9, apoC3 and sdLDL-C with current dyslipidemias, to investigate the roles of these markers in determining potential lipid disorders.

The principal findings of the present study were threefold. First, in the cohort of patients who were not taking a lipid-lowering drug before the first CAG, we showed that lipid disorders were associated with multiple parameters of lipid and lipoproteins, and the novel lipid measurements such as PCSK9, apoC3, and sdLDL-C seemed to play important roles. Second, we investigated the interactions of the novel measurements with current dyslipidemias and identified the levels for predicting current dyslipidemias, respectively. Specially, PCSK9 was associated significantly with hypercholesterolemia or combined hyperlipidemia; apoC3 was significant in hypertriglyceridemia, hypercholesterolemia, and combined hyperlipidemia; and sdLDL-C was highlighted in all current dyslipidemias classification. Third, we evaluated the discordances of PCSK9, apoC3, and sdLDL-C with current dyslipidemias, and found substantial discordances exist in each dylipidemia group and normalipidemia patients. These findings (although observational) might highlight the clinical interest of the novel lipid factors in determining potential lipid disorders and future cardiovascular risk, and provide novel view in lipid management and cardiovascular benefit in future medicine.

PCSK9 is well-recognized as a circulatory ligand that terminates the lifecycle of the LDL receptor (LDLR) thus affecting circulating LDL-C levels [10]. Humans with loss-of-function of PCSK9 have extremely low levels of LDL-C, and even small LDL-C reduction due to common mutations in PCSK9 have been shown to reduce lifetime cardiovascular events (CVEs) [11]. Moreover, anti-PCSK9 therapies are exceptionally effective in lowering LDL-C levels and CVEs [12]. Recent comprehensive reviews have summarized the history of PCSK9 and the relation to LDL-C and CVD [7, 13]. Similarly, the well connection was also made by apoC3, with triglycerides and CVD [8, 14, 15]. ApoC3 is a small protein found in both the postprandial and fasting state on chylomicrons, very LDL (VLDL), and HDL [14]. It was first described as an inhibitor of lipoprotein lipase (LPL), but it also interferes with clearance of triglyceride-rich lipoprotein (TRL) remnants by the LDLR in the liver [14]. ApoC3 also promotes the synthesis and secretion of TRL and modulates intestinal lipid absorption [14]. Moreover, genetic loss-of-function of APOC3 is associated with lower triglycerides and a reduced risk of CVD [8]. In fact, LDL is a heterogeneous group of particles differing not only in size and density but also in chemical composition and physiological function. Different lipoprotein subfractions play varied role in the pathogenesis of CVD. Although recent studies have demonstrated that the content of LDL-C in LDL particles displays a large inter-individual variation, the measurement of sdLDL-C correlate more strongly with cardiovascular risk than other LDL-C and large LDL particle concentrations [16].

Although prior studies have explored possible correlations of PCSK9 [17], apoC3 [15], and sdLDL-C [18] with lipid profile, including TG, LDL-C, and HDL-C, and their role in atherogenisis, few of them assessed the role of these parameters in determining current dyslipidemias and compared the discordance. The present study suggested patients who had high PCSK9, apoC3, or sdLDL-C might need aggressive lipid management. Notwithstanding the effectiveness of lowering LDL-C, residual CVD risk remains in high-risk populations, likely contributed to by non-LDL-C lipid abnormalities [4]. Our data might mark an epidemic of the novel atherogenic lipid measurements, and their associations with current dyslipidemias including hypertriglyceridemia, hypercholesterolemia, combined hyperlipidemia, and hypo HDL cholesterolemia.

Nevertheless, there were several limitations of the present study. First, the study was with cross-sectional nature, indicating only association, but not causality. Prospective study with a long follow-up for predicting dyslipidemia and CVD might strengthen the results. Second, we just strictly limited the use of lipid-lowering drugs from the enrollment. The anti-diabetic and anti-hypertensive drugs used by the participants had not collected in the starting point although they had not been started the second-level prevention from a cardiologist yet. Finally, the observed associations might not be generalizable across races, the study population was the Chinese population from a single center.

In conclusion, PCSK9, apoC3, and sdLDL-C showed significant interactions with current dyslipidemias, and were predictive in the screening. The substantial discordances with current dyslipidemias might provide novel view in lipid management and cardiovascular benefit.

## MATERIALS AND METHODS

### Study population

Our study complied with the Declaration of Helsinki and was approved by the hospital's ethical review board (Fu Wai Hospital & National Center for Cardiovascular Diseases, Beijing, China). Informed written consent was obtained from all patients enrolled in this study.

To study the associations of PCSK9, apoC3, and sdLDL-C with current dyslipiemias, we enrolled the patients with no lipid-lowering treatment at least 3 months prior to entering the study from the patients scheduled for diagnostic/interventional CAG because of their first angina-like chest pain and/or positive treadmill exercise test or clinically suspected CVD in our division as our previous studies [16, 19]. Therefore, we recruited the study patients with angiography and lipid-lowering-therapy being parts of the screening process. Patients with acute coronary syndrome (ACS), heart failure (left ventricular ejection fraction, LVEF < 45%), significant hematologic disorders, infectious or systematic inflammatory disease, thyroid dysfunction, severe liver and/or renal insufficiency and malignant disease were excluded from the current study. Finally, the cross-sectional study comprised 1605 consecutive patients for analysis.

Patients were classified into five groups according to current dyslipidemias classification [20]: hypertriglyceridemia (*n* = 359) was defined according to cut-points of TG > 1.7 mmol/L but TC≤5.18 mmol/L; hypercholesterolemia (*n* = 250), which was included with patients of TC > 5.18 mmol/L but TG≤1.7mmol/L; combined hyperlipidemia (*n* = 312), was defined as both TG > 1.7 and TC > 5.18 mmol/L; hypo HDL cholesterolemia was consisted with patients with HDL-C < 1.0mmol/L (*n* = 323); and normalipidemia (*n* = 361), was the patients with normal levels of TG, TC, and HDL-C.

### Laboratory examinations

All patients underwent clinical examination and blood testing as our previous studies [16, 19]. Blood samples were obtained for all patients from the cubital vein after a 12-hour overnight fast.

The concentrations of the plasma TC, TG, HDL-C, LDL-C, apoAI, apoB were measured using an automatic biochemistry analyzer (Hitachi 7150, Tokyo, Japan), and the TC, TG, HDL-C, and LDL-C levels were measured using an enzymatic assay. ApoAI, apoB levels were measured using a turbidimetric immunoassay. HbA1C was measured using Tosoh Automated Glycohemoglobin Analyzer (HLC-723G8, Tokyo, Japan). The concentrations of hs-CRP were determined using immunoturbidimetry (Beckmann Assay 360, Bera, Calif., USA).

Plasma PCSK9 levels were measured by high-sensitivity, quantitative sandwich enzyme measured by a high sensitivity, quantitative sandwich enzyme-linked immunosorbent assay using CircuLex ELISA kit, which incorporated an antibody specific for PCSK9, The sensitivity of the assay was 0.154 ng/ml. Plasma apoC3 levels were measured using the RayBio^®^ ELISA kit, which is an *in vitro* enzymelinked immunosorbent assay for the quantitative measurement and employs an antibody specific for human ApoC3. The minimum detectable dose of Human ApoC3 was determined to be 2.5 pg/ml. sdLDL-C analysis was performed electrophoretically by the use of high-resolution 3% polyacrylamide gel tubes and the Lipoprint LDL System (Quantimetrix Corporation, Redondo Beach, CA, USA) according to the manufacturer's instructions as previously described. LDL was divided in 7 subfractions by this analysis, and the subfractions of 3 to 7 are grouped into the sdLDL subclass.

### Statistical analysis

The values were expressed as the mean ± SD and the number (percentage) for the categorical variables. The differences of clinical and biochemical parameters between groups were analyzed using analysis of variance, and χ2-tests where appropriate. A general linear model was used to study the lipid measurements over the groups of dyslipidemias, with adjustments for confounding factors including age, gender, BMI, hypertension, HbA1C, smoking, hs-CRP, and coronary statues, and to calculate the adjusted mean values. Because PCSK9, apoC3, and sdLDL-C pathogenicity increases significantly at high levels, we performed polytomous logistic regression analysis to evaluate the association between the high values according to the cut-points from the analysis of receiver-operating characteristic curves to explore the strength of the interactions with current dyslipidemias. OR and 95% CI was calculated with the logistic models adjusted by potential confounding factors such as age, gender, BMI, hypertension, HbA1C, smoking, hs-CRP, dyslipidemias classification, and coronary status. A *p*-value < 0.05 was considered statistically significant. The statistical analysis was performed with SPSS version 19.0 software (SPSS Inc., Chicago, IL, USA).

## SUPPLEMENTARY MATERIALS FIGURES


