# Supplementary material for: Novel circulating lipid measurements for current dyslipidemias in non-treated patients undergoing coronary angiography: PCSK9, apoC3 and sdLDL-C
Source: Oncotarget. 2016 Oct 4;8(7):12333–41. doi: 10.18632/oncotarget.12471 (PMC5355348; doi:10.18632/oncotarget.12471)
Supplement: Supplementary file 1 [file oncotarget-08-12333-s001.pdf]

Novel circulating lipid measurements for current dyslipidemias in non-treated patients undergoing coronary angiography: PCSK9, apoC3 and sdLDL-C

Supplementary Material

Supplemental Table. Relationships between the circulating lipid measurements and the results of coronary angiography

Angiographic Results	PCSK9 (ng/ml)	ApoC3 (µg/ml)	sdLDL-C (mg/dl)
Negative (n=588)	231.35±69.34	90.64±51.70	8.05±10.04
Positive (n=1017)	238.87±68.58	99.54±63.76	8.90±9.64
P-value	0.245	**0.023**	**0.025**
Diseased coronary branches			
LM-involved (vs. negative)	236.05±68.38	**104.24±50.56**	8.09±8.15
LAD-involved (vs. negative)	238.64±68.92	97.88±61.27	**8.93±9.70**
LCX-involved (vs. negative)	242.58±68.92	97.57±68.89	**9.14±9.26**
RCA-involved (vs. negative)	238.86±67.46	**102.70±64.65**	**8.97±9.69**
Number of diseased vessels			
1-vessel (n=334)	234.22±65.39	94.16±61.26	8.41±9.46
2-vessel (n=285)	239.76±65.89	108.57±46.57	9.18±9.42
Multi-vessel (n=398)	239.83±70.21	98.73±48.06	8.95±9.62
P-value	0.428	0.676	0.531

Data shown are mean ± SD. The bold values indicate statistical significance and are bolded to improve the readability of the table. LM, left major coronary artery; LAD, left anterior descending artery; LCX, left circumflex artery; RCA, right coronary artery. 1-vessel, 1 diseased vessel in one of LM, LAD, LCX, or RCA; 2-vessel, 2 diseased vessels in two of LM, LAD, LCX, or RCA; multi-vessel, 3 or 4 diseased vessels from LM, LAD, LCX, or RCA.
